# Training and race-induced coordination of oxidative stress markers in racehorses: insights from multivariate and univariate analyses

**DOI:** 10.1093/jvimsj/aalaf085

**Published:** 2026-02-03

**Authors:** Maciej Kacprzyk, Izabela Dąbrowska, Jowita Grzędzicka, Dominika Milczek-Haduch, Paula Kiełbik, Marcin Gołębiewski, Olga Witkowska-Piłaszewicz

**Affiliations:** Institute of Animal Sciences, Warsaw University of Life Sciences, Department of Animal Breeding, Warsaw 02-787, Poland; Institute of Veterinary Medicine, Warsaw University of Life Sciences, Department of Large Animal Diseases and Clinic, Warsaw 02-787, Poland; Institute of Veterinary Medicine, Warsaw University of Life Sciences, Department of Large Animal Diseases and Clinic, Warsaw 02-787, Poland; Institute of Veterinary Medicine, Warsaw University of Life Sciences, Department of Large Animal Diseases and Clinic, Warsaw 02-787, Poland; Institute of Veterinary Medicine, Warsaw University of Life Sciences, Department of Large Animal Diseases and Clinic, Warsaw 02-787, Poland; Institute of Veterinary Medicine, Warsaw University of Life Sciences, Department of Large Animal Diseases and Clinic, Warsaw 02-787, Poland; Institute of Animal Sciences, Warsaw University of Life Sciences, Department of Animal Breeding, Warsaw 02-787, Poland; Institute of Veterinary Medicine, Warsaw University of Life Sciences, Department of Large Animal Diseases and Clinic, Warsaw 02-787, Poland

**Keywords:** SOD, TBARS, total antioxidant capacity, exercise physiology, redox adaptation, oxidative stress

## Abstract

**Background:**

Oxidative stress is a major physiological challenge in racehorses and may be modulated through training-induced adaptation.

**Hypothesis/Objectives:**

Repeated exercise will induce not only biomarker-specific changes but also will enhance coordination between oxidative and antioxidative responses in racehorses.

**Animals:**

Thirty-one clinically healthy racehorses (9 Thoroughbreds, 22 Arabians; 12 mares, 19 stallions) from a single training center.

**Methods:**

Prospective cohort study. Serum concentrations of oxidative stress markers (advanced oxidation protein products [AOPPs], superoxide dismutase [SOD], total antioxidant capacity [TAOC], and thiobarbituric acid reactive substances [TBARS]) were measured by enzyme-linked immunosorbent assay before and 30 min after standardized exercise at 3 time points: early training (T1), post-training (T2), and after a race (R). Univariate analysis assessed fold changes (FC), principal component analysis (PCA), and hierarchical clustering were used to examine inter-marker coordination. The primary outcome was systemic redox adaptation over time.

**Results:**

Post-exercise, AOPP, and SOD increased (median log-fold change, 0.13 and 0.05; approximately 1.14× and 1.05×; *P* = .03 and *P* = .01), whereas TBARS and TAOC showed no significant univariate changes. Superoxide dismutase responses were larger after races than after training (*P* < .05). Principal component analysis and correlation matrices identified enhanced post-exercise coordination among TBARS, SOD and TAOC (eg, TBARS-SOD r = 071), whereas AOPP remained weakly correlated, consistent with distinct regulatory dynamics.

**Conclusions and clinical importance:**

Structured training and racing were associated with a post-exercise reorganization of the redox profile (tighter coupling among SOD, TAOC and TBARS) with AOPP remaining relatively independent. These data strengthen existing evidence for training-related redox adaptation and highlight the added value of multivariate analysis for capturing inter-marker coordination beyond univariate trends.

## Introduction

Oxidative stress, resulting from an imbalance between reactive oxygen species (ROS) production and antioxidant defenses, is a key physiological phenomenon in equine athletes subjected to high-intensity exertion. Acute exercise can induce lipid peroxidation and protein oxidation, which are commonly measured using biomarkers such as thiobarbituric acid reactive substances (TBARS) and advanced oxidation protein products (AOPP), respectively. Whereas AOPP are widely used and validated for matrices in horses, they capture a specific subset of protein modification. Therefore, they should be interpreted alongside broader indices of protein oxidation such as protein carbonyl content, dityrosine, and thiol/disulfide redox status to provide a balanced view of redox alterations.^1,2^ Concurrently, enzymatic and non-enzymatic antioxidant responses represented by superoxide dismutase (SOD) and total antioxidant capacity (TAOC) can be upregulated to restore redox homeostasis.^3–5^ Previous studies have shown that antioxidant responses are mobilized and can be upregulated or maintained at high levels. However, their direction and magnitude are strongly dependent on the exercise protocol and may, in some cases, result in measurable depletion or downregulation of certain markers.^6–8^ These findings emphasize the complex interplay between oxidative challenges and physiological adaptation in sport horses.

Despite growing interest in redox biology, current understanding of systemic oxidative stress adaptation in racehorses remains limited. Most existing research focuses on univariate assessment of individual biomarkers after isolated exercise bouts, often overlooking the long-term effects of repeated training on redox dynamics. Moreover, such studies rarely investigate the relationships between biomarkers or assess whether training promotes improved inter-marker coordination. Notably, AOPP has been shown to follow regulatory dynamics distinct from lipid peroxidation markers such as TBARS or enzymatic antioxidants such as SOD.^4,9^ The lack of multivariate approaches in equine exercise physiology represents an important gap because complex biological responses to training may manifest not in large changes in single markers, but in more subtle, systemic reorganizations detectable only using advanced statistical methods such as principal component analysis (PCA) or hierarchical clustering (HC).^10,11^ Although redox balance is sometimes approximated by ratio-type indices (eg, pro-oxidant to antioxidant markers),^12–14^ such univariate composites can be scale-dependent and may obscure the underlying covariance structure that reflects biological coordination. We therefore prioritized multivariate analyses (correlation matrices, PCA, HC) to quantify inter-marker co-regulation, and interpreted univariate changes in the context of this systemic structure. We hypothesized that race training in horses would induce oxidative adaptation not primarily through major shifts in individual oxidative stress or antioxidant markers, but through enhanced coordination among them. Our objective was 2-fold: to quantify intra-individual changes in 4 oxidative stress-related biomarkers (AOPP, SOD, TAOC, TBARS) across three exercise contexts (early training, after training, racing) and evaluate whether training improves systemic oxidative coordination using multivariate analyses. By assessing dynamic inter-marker relationships in the same cohort over time, we aimed to offer novel insights into redox regulation and adaptive responses in elite racehorses.

## Material and methods

### Animals

Our study was conducted on 9 Thoroughbred and 22 Arabian racehorses (*n* = 31), clinically healthy and actively participating in race training. The horses included 12 mares and 19 stallions aged 2-7 years, all housed at a single training facility under the guidance of one professional trainer. Each horse was kept in an individual stall with appropriate bedding and had training sessions under saddle 6 times per week when weather conditions allowed. We enrolled Thoroughbred and Arabian racehorses that were in active race training at a single professional yard under one trainer; clinically healthy based on physical examination and routine hematology and biochemistry, managed under uniform housing and centralized feeding (identical hay and one commercial concentrate with a single vitamin–mineral premix; no other supplements permitted), and with routine selenium screening to detect deficiency or excess.

Thus, feeding was centralized and uniform across the cohort: the same high-quality hay and a single commercial concentrate with one vitamin–mineral premix were provided; rations were adjusted to body weight and day-to-day workload according to the yard’s protocol; no supplements beyond the premix were permitted. Selenium status was assessed using a routine veterinary panel to screen for deficiency or excess.

The average body weight of the horses was approximately 450-550 kg. Training intensity was individually adjusted based on routine assessments of cardiovascular fitness, including heart rate monitoring, speed monitoring and hematologic and biochemical variables, and blood lactate measurements performed during standardized exercise tests. All horses were clinically healthy based on clinical examination (temperature, heart rate, respiratory rate, and absence of nasal discharge, cough, colic signs, and lameness) and hematologic evaluation, dewormed and vaccinated before the racing season, with no medications or non-protocol supplements administered in the preceding period. Pre-training conditions on sampling days were identical for all horses. No randomization or performance-based pre-selection was applied; breed and sex distribution reflected the yard’s composition.

### Training protocol

The horses underwent a structured race training program, with intensity adjusted according to the training day. The schedule included: warm-up: 10 min of walking under the rider, followed by 800 m of trotting and 800 m of slow (hack) canter, conditioning phase: 1400-2400 m canter at a moderate pace (16-22 s per 200 m), high-intensity phase: full gallop sessions on 500-1200 m at 80%-100% of maximal speed (13-15 s per 200 m), cool-down: 30-40 min of walking on a mechanical walker. Intensive gallop sessions were included every 5-10 days to enhance race performance.

Blood Sampling Protocol.

Blood samples were collected before (p0) and 30 min (p1) after exercise at multiple time points to assess both baseline and exercise-induced physiological responses:

- At the beginning of the training season (T1)
- After 6-10 weeks of training (T2)
- After flat races 1600-2200 m (R)
- Before exercise and 30-40 min after exercise in T1/T2/R.

Baseline (p0) venous blood was collected in the morning, before feeding, and exercise. Horses had access to hay overnight; no concentrates or supplements were offered after the previous evening meal, and no feed was provided on the sampling morning before phlebotomy; water was available ad libitum. No medications or non-protocol supplements were administered for ≥3 weeks before sampling, and none were given immediately before p0. We deliberately selected the 30-min post-exercise point (p1) to capture the early phase of the systemic response, which according to equine literature persists for at least 30 min after intensive exercise.^5,14,15^

Venous blood was collected from the jugular vein using sterile needles into EDTA-coated tubes for hematologic analysis and into plain tubes for serum biochemistry. All blood sampling procedures were carried out as part of routine veterinary monitoring and exercise assessments. As per European Directive EU/2010/63 and Polish regulations on animal experimentation, ethical approval was not required because these procedures were classified as non-experimental clinical veterinary practices.

Heart rate, speed during standardized exercise tests, and lactate were used operationally to tailor training loads; these variables were not analyzed as predictors of the redox profile and were not included in multivariate models.

### Laboratory assays

Serum samples were separated by centrifugation (3000×*g*, 10 min, 4 °C) and stored at −80 °C until analysis. The concentrations of AOPP, SOD, TAOC, and TBARS were measured using commercially available enzyme-linked immunosorbent assay (ELISA) kits (Wuhan Xinqidi Biological Technology Co., Ltd.), following the manufacturers’ protocols. All assays were optimized for equine serum and exhibited acceptable precision and sensitivity parameters in accordance with manufacturer specifications and prior animals’ applications. Analytical precision of each ELISA was assessed using serum measured in quintuplicate at low, medium, and high concentrations. Intra-assay coefficients of variation were calculated as SD/mean × 100 and are provided in Table S3.

The ELISA assays used in this study demonstrated the following precision levels:

- AOPP ELISA test: Detection range: 10-0.156 ng/mL, sensitivity: up to 0.05 ng/mL, intra-assay precision: ≤8%, inter-assay precision: ≤12%
- SOD ELISA test: Detection range: 500-7.8 ng/mL, sensitivity: up to 1 ng/mL, intra-assay precision: ≤8%, inter-assay precision: ≤12%
- TAOC ELISA test: Detection range: 1000-15.6 U/mL, sensitivity: up to 5 U/mL, intra-assay precision: ≤8%, inter-assay precision: ≤12%
- TBARS ELISA test: Detection range: 20-0.312 ng/mL, Sensitivity: up to 0.06 ng/mL, intra-assay precision: ≤8%, inter-assay precision: ≤12%.

### Data analysis

We focused on systemic redox coordination. We did not correlate biomarker changes with finishing position or official ratings, because the cohort spanned 2 breeds, variable field conditions, and sample size constraints that could bias under-powered models. Data analysis included a cohort of racehorse samples (*n* = 77) collected before and 30 min after standardized training sessions (T1 and T2) and races (R). Data distribution normality was evaluated using the Shapiro–Wilk statistical test and screened for outliers for each biomarker using the Grubbs method.

To statistically evaluate the combined influence of sex (mare vs. stallion), breed (Thoroughbred vs. Arabian), and training stage (T1, T2, R) on the relative changes (fold change, FC) of oxidative stress biomarkers, a supervised three-way multivariate analysis of variance (MANOVA) was performed. The multivariate response variables included FC values of AOPP, SOD, TAOC, and TBARS. The MANOVA was conducted using Python (statsmodels library, version 3.9). The full factorial model included: 3 main effects (sex, breed, training type), 3 two-way interactions (sex × breed, sex × training type, breed × training type), 1 three-way interaction (sex × breed × training type). Assumptions of multivariate normality and homogeneity of variance–covariance matrices were assessed before analysis. Multivariate test statistics reported included Wilks' lambda, Pillai's trace, Hotelling-Lawley trace, and Roy’s greatest root, with Wilks’ lambda primarily used for interpretation. Statistical significance was determined at *P* < .05. Although no significant effects were found, all model outputs were retained to ensure analytical transparency and are presented in the Table S2. Multivariate approaches such as unsupervised PCA were chosen to complement univariate FC analysis by capturing underlying systemic adaptations that may not be evident using individual marker testing alone.

All statistical analyses, except three-way MANOVA, including univariate tests, FC computations, PCA, and HC were performed using OriginPro 2022 (OriginLab Corporation, Northampton, MA, USA).

### Univariate analysis

Relative FCs for each oxidative stress marker (AOPP, SOD, TAOC, TBARS) were calculated as the ratio of after training concentrations to before training baseline concentrations for each horse. The FC values were statistically evaluated for significant deviations from a theoretical median of zero (indicating no change) using either the one-sample Wilcoxon signed rank test or the one-sample *t* test, depending on data normality distribution verified using the Shapiro–Wilk test. Statistical significance was set at *P* < .05.

### Multivariate analysis

To explore inter-marker relationships and underlying patterns of oxidative stress response, unsupervised PCA was applied separately to before and after exercise datasets. It was performed on correlation matrices derived from *z*-standardized values, ensuring that each biomarker contributed equally, regardless of scale. Standardization was done by subtracting the variable mean and dividing by its SD (*z*-score transformation), allowing for meaningful comparison of markers with different units or variability. Principal component analysis was performed separately for the pooled before datasets and pooled after datasets (ie, across T1/T2/R within each p-layer) and in an integrated analysis combining before and after to visualize the global reorganization of the redox profile with exercise. We did not compute PCA separately within each of T1, T2, and R because of stability and power considerations.

Correlation coefficients (*r*) were interpreted using the following thresholds: |*r*| < 0.3 (weak), 0.3 ≤ |*r*| < 0.7 (moderate), and |*r*| ≥ 0.7 (strong), in accordance with commonly accepted conventions in biomedical research.^8,9^

To complement the PCA results and provide an alternative visualization of inter-marker relationships, unsupervised HC analysis was conducted using the same *z*-standardized datasets. The clustering was based on Euclidean distance and the Ward linkage method, which minimizes variance within clusters. Hierarchical clustering was applied separately to before exercise, after exercise, and combined datasets. Dendrograms were generated to illustrate similarity patterns between oxidative stress markers and to investigate how exercise influenced the systemic organization of redox responses.

## Results

Across T1, T2, and R, baseline (p0) concentrations of AOPP, SOD, TAOC, and TBARS remained within routine accepted values, indicating that training and racing protocol did not induce deviations at rest. Instead, the principal adaptive signal was expressed as post-exercise (p1) changes in relationships between the markers, as captured by PCA and HC.

**Figure 1:**
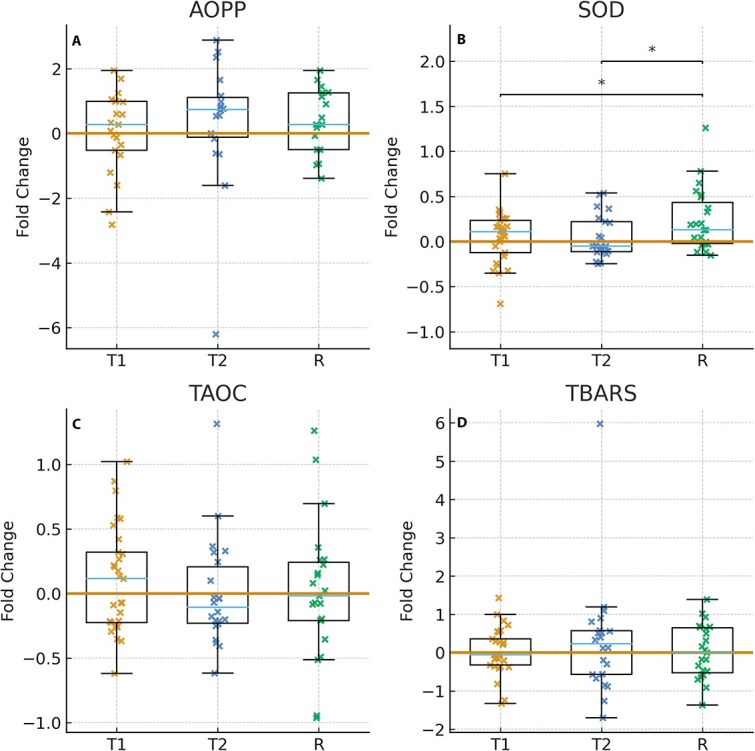
Fold change in oxidative stress markers—AOPP, SOD, TAOC, and TBARS—After exposure to different exercise contexts: Training 1 (T1), training 2 (T2), and a race (R). Each panel (A–D) represents the marker-specific fold change relative to the pre-exercise baseline. Half-boxplots display the interquartile range (IQR 25th-75th percentile) with the median indicated by a central line and whiskers extending to 1.5 times the IQR. Individual data points are presented on the right side of the box. Significant differences between conditions are indicated with asterisks (^*^P < .05).

To evaluate redox balance in response to different physical workloads, FC in oxidative stress markers across 2 training sessions (T1 and T2) and a race (R) were compared (Figure 1). Advanced oxidation protein products, TAOC, and TBARS (panels A, C, and D) showed no significant differences between exercise types. In contrast, SOD activity (panel B) significantly increased after the race compared with both training sessions (R vs. T1 *P* = .04; R vs. T2 *P* = .04). No significant differences were detected between T1 and T2.

### FC analysis

**Figure 2:**
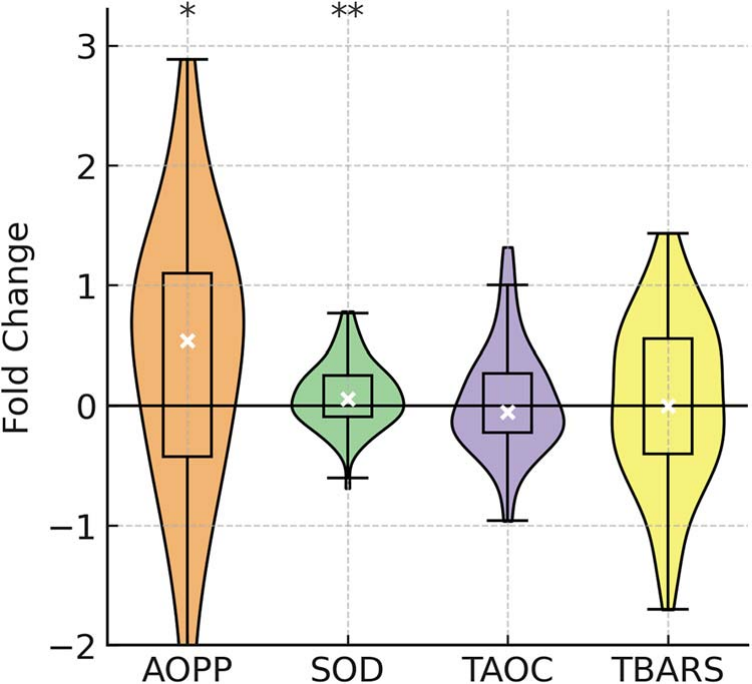
Distribution of fold change (FC) values for each oxidative stress marker (AOPP, SOD, TAOC, TBARS) in racehorses (after relative to before exercise). The violin plot shows the FC, where 0 indicates no change. The box within the violin plot presents a 25%-75% interquartile range (IQR) with a median (white circle) and whiskers as a range within 1.5 IQR. Significant changes from 0 are indicated with asterisks (^*^P < .05; ^**^P < .01; ^***^P < .001).

Violin plots illustrating FC indicated no significant alterations in the concentrations of TAOC or TBARS after exercise. However, the substantial increase in concentration of AOPP (median FC, 0.51; *P* = .03) and SOD (median FC, 0.06; *P* = .001) was identified (Figure 2).

### Principal component analysis

**Figure 3:**
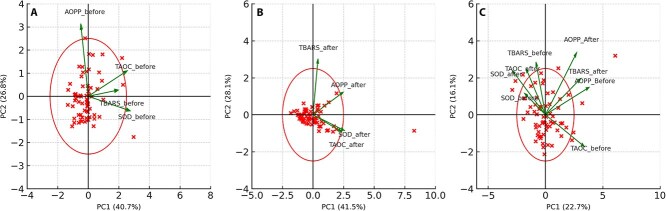
Principal component analysis (PCA) of *z*-standardized concentration of oxidative stress markers before and after exercise. PCA biplot of standardized oxidative stress markers before exercise (A), after exercise (B), and combined PCA of both before and after exercise markers (C).

Principal component analysis of oxidative stress markers before exercise identified 2 primary components explaining 40.7% (PC1) and 26.8% (PC2) of the total variance, respectively (Figure 3A). Consistently, the correlation matrix showed a strong positive correlation between SOD and TAOC (*r* = 0.67), and moderate positive correlations between TBARS and both SOD (*r* = 0.56) and TAOC (*r* = 0.60; Table 1). After exercise, PCA similarly identified 2 components accounting for 41.5% (PC1) and 28.1% (PC2) of variance (Figure 3B). The correlation matrix showed that SOD and TAOC remained moderately correlated (*r* = 0.55), whereas correlations between TBARS and antioxidant markers were weak (SOD–TBARS *r* = −0.05; TAOC–TBARS *r* = −0.03). AOPP (after) demonstrated a moderate positive correlation with SOD (after; *r* = 0.36), whereas its associations with TAOC and TBARS remained weak (Table 2).

**Table 1:** Correlation matrix of oxidative stress markers (*z*-standardized) sampled before exercise.

	**AOPP (before)**	**SOD (before)**	**TAOC (before)**	**TBARS (before)**
**AOPP (before)**	1	−0.19	0.10	−0.02
**SOD (before)**	−0.19	1	0.67	0.56
**TAOC (before)**	0.10	0.67	1	0.60
**TBARS (before)**	−0.02	0.56	0.60	1

**Table 2:** Correlation matrix of oxidative stress markers (*z*-standardized) after exercise.

	**AOPP (after)**	**SOD (after)**	**TAOC (after)**	**TBARS (after)**
**AOPP (after)**	1	0.36	0.26	0.23
**SOD (after)**	0.36	1	0.55	−0.05
**TAOC (after)**	0.26	0.55	1	−0.03
**TBARS (after)**	0.23	−0.05	−0.03	1

No distinctive clustering by sex, breed, or response pattern was evident in PCA biplots, suggesting homogeneity in oxidative marker response among horses after standard training procedures (Figure S1).

To better understand systemic shifts induced by exercise, a combined PCA including both before and after exercise markers was performed (Figure 3C). This integrated analysis identified a global reorganization of the oxidative stress profile, with PC1 accounting for 22.7% and PC2 for 16.1% of total variance. Antioxidant markers and TBARS (before) clustered closely together, reflecting improved inter-marker coordination after training. In contrast, AOPP (before) and AOPP (after) vectors remained directionally isolated, supporting their role as independently regulated components of the redox system and together with TBARS (after), occupied a separate region of the biplot. Notably, TBARS (after) shifted closer to the origin, indicating decreased individual variation and suggesting a more uniform oxidative response after exercise.

### Hierarchical clustering

**Figure 4:**
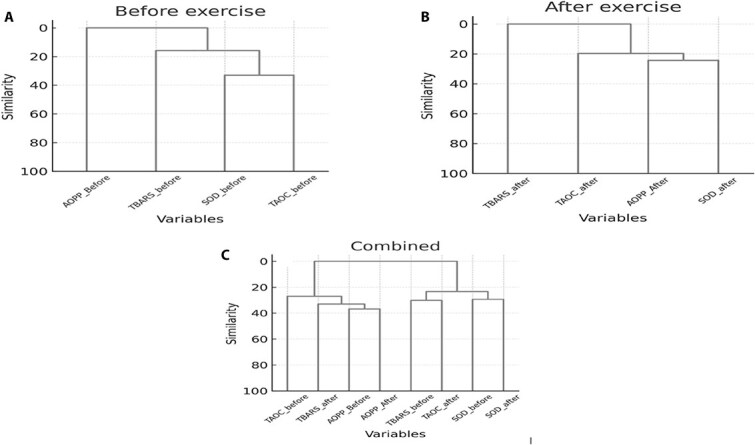
Hierarchical clustering dendrograms of oxidative stress markers measured (A) before exercise, (B) after exercise, and (C) combined across both time points. Clustering was performed on *z*-standardized marker values using Euclidean distance and Ward’s minimum-variance linkage. Each branch represents the relative similarity between variables, with lower branch heights indicating more similarity.

To further explore inter-variable relationships, HC was applied to the standardized oxidative stress markers before and after exercise (Figure 4A and B). The resulting dendrograms for both time points had a very similar structure, with consistent grouping of antioxidant markers (SOD and TAOC), and TBARS (before) joined this antioxidant/lipid-peroxidation module at a slightly lower similarity, and AOPP (before) formed the most distant branch. Advanced oxidation protein products (after) moved closer to antioxidant cluster, and TBARS (after) became the most isolated marker.

In the combined analysis (Figure 4C), the temporal shifts in marker relationships became more apparent. AOPP (before) and AOPP (after) clustered together but distinctly apart from all other markers, emphasizing their persistent independence from redox dynamics seen in TBARS, SOD, and TAOC.

In the combined dendrogram, SOD and TAOC from both time points formed a tight cluster together with TBARS (before), whereas AOPP (before), AOPP (after), and TBARS (after) formed a separate branch. Hierarchical clustering therefore complements the PCA results by confirming the stability of the SOD/TAOC core and highlighting the context-dependent behavior of TBARS and the relatively independent position of AOPP within the redox network.

## Discussion

We demonstrated that structured race training in horses does not cause large shifts in the concentrations of oxidative stress biomarkers such as TBARS, TAOC, or SOD, with the exception of a moderate increase in AOPP and after race SOD activity. However, multivariate analyses identified an exercise dependent reorganization of relationships among antioxidant and lipid peroxidation markers after exercise, with SOD and TAOC forming a stable core module and TBARS and AOPP showing more independent behavior after exercise. These findings align with the concept that race-trained equine athletes develop more efficient regulatory responses to oxidative challenges, maintaining homeostasis through subtle inter-marker adjustments rather than dramatic changes in absolute biomarker concentrations. Objective yard fitness monitoring (heart rate, speed, hematology, biochemistry, and lactate) was used operationally to adjust training and confirm readiness, but these data were not prespecified study endpoints and are therefore not analyzed here.

The most significant univariate changes observed were increases in AOPP and SOD concentrations after exercise, consistent with previous reports indicating that physical activity triggers acute oxidative stress and antioxidant defense mechanisms.^3,4^ Specifically, SOD activity increased after races compared with training sessions, suggesting that the more intense physical exertion of competition elicits a stronger enzymatic antioxidant response. This response may reflect both higher ROS production and enhanced capacity for enzymatic neutralization after repeated training stimuli.^16,17^ The increase in AOPP across all exercise contexts implies that protein oxidation is sensitive to physical exertion regardless of its intensity, although its behavior differs markedly from other redox markers.

The PCA yielded an important and novel finding. Before exercise, TBARS was moderately and positively correlated with both SOD and TAOC whereas after exercise, measurements weakened to near zero. This pattern suggests that the acute post-exercise response is dominated by a tightly coordinated SOD/TAOC module, whereas TBARS becomes more weakly coupled and reflects a partly independent lipid peroxidation process. This tight clustering suggests an improved, integrated systemic response to oxidative stress, which is a hallmark of physiological adaptation.^18,19^ Horses that experienced larger oxidative challenge, as indicated by increased TBARS, also mounted stronger antioxidant responses, reflected in higher SOD and TAOC. Conversely, those with minor increases in lipid peroxidation also showed only modest antioxidant activation. This coupling may reflect effective feedback and regulatory mechanisms strengthened through training.

Notably, AOPP remained weakly correlated with other markers across all analyses. Principal component analysis, HC, and correlation matrices showed stronger clustering of TAOC and SOD after training, whereas the association of TBARS with this antioxidant core was context-dependent (moderate before exercise but much weaker afterward), whereas AOPP vectors remained directionally isolated. This observation is consistent with findings that protein oxidation may follow distinct regulatory kinetics or reflect more stable, cumulative processes compared with the dynamic changes in lipid peroxidation and enzymatic antioxidant activity.^4,10^ In jumper horses, basal AOPP concentrations were significantly correlated with total oxidant status and nitric oxide metabolites, suggesting that protein oxidation is regulated by broader redox or inflammatory pathways rather than acute exercise itself.^4^

The observed dissociation between AOPP and other redox markers also may reflect differences in molecular targets and repair mechanisms. Lipid peroxidation products such as TBARS are typically cleared rapidly, whereas advanced oxidation protein products may accumulate over time and reflect chronic oxidative damage or inflammatory activity.^1,20^ Our findings suggest that whereas TBARS, SOD, and TAOC constitute a responsive, coordinated arm of the acute redox response, AOPP may represent a slower, perhaps more structurally impactful, oxidative process.

The multivariate findings are of particular importance. In many previous studies, oxidative stress in horses has been assessed using individual markers, leading to sometimes inconsistent or incomplete conclusions. Here, the use of PCA and HC identified hidden relationships and systemic adaptations that would not be evident from univariate analysis alone. After training, PCA showed a reduction in inter-individual variation and clustering of antioxidant and lipid oxidation markers, which is an evidence that training reorganizes rather than uniformly strengthens the correlations among markers: a stable SOD/TAOC axis is maintained, whereas TBARS and AOPP adopt condition dependent positions relative to this core. These insights emphasize the value of multivariate approaches in capturing complex biological adaptation to training stimuli.^10,11^

In this field cohort of race-trained horses managed by a single trainer, multivariate analyses indicated a stable SOD/TAOC module with condition-dependent positioning of TBARS and relatively decoupled AOPP. We interpret these findings as state-dependent organization within this dataset, not as evidence that redox coordination is a universal marker of adaptation independent of demographics. Prior studies of horses reported breed-, sex-, and age-related differences in oxidant/antioxidant status, cautioning against broad extrapolation.^21–23^ In this single yard, mixed breed cohort, three-way MANOVA detected no significant multivariate effects of sex, breed, or their interactions with exercise context on the vector of oxidative/antioxidative changes (all Wilks’ lambda *P* > .05; Table S2), but precision is limited. Larger, prespecified studies are needed to test whether coordination metrics generalize across populations. The concept of oxidative hormesis may help explain these findings. Exercise-induced ROS production, although potentially damaging at high levels, also plays a crucial role in promoting beneficial adaptive responses, including upregulation of antioxidant enzymes and mitochondrial biogenesis.^24,25^ In this context, the reorganization among oxidative stress markers relationships seen in our study can be interpreted as a result of repeated low level redox challenge during training, one that primes the physiological system for more efficient responses during future exercise bouts. This conclusion is supported by similar findings in human athletes, where regular physical activity leads to decreased resting oxidative damage and improved redox regulation.^26,27^

Physiologically, the context dependent coupling between TBARS relative to the stable SOD/TAOC axis observed after exercise implies that trained horses are better equipped to manage the oxidative challenge of racing without accumulating long-term damage. This systemic adaptation was confirmed by the fact that none of the horses exhibited injury or performance issues during or after the study period. These findings are relevant for performance monitoring and could inform individualized conditioning programs based on oxidative stress profiles.

From a practical standpoint, our results suggest that evaluating systemic coordination of redox markers may provide more clinically relevant insight than assessing isolated biomarker results. For example, a horse with stable TBARS values but uncoordinated SOD responses may be more vulnerable to redox imbalance than a horse showing moderate, well-matched changes in all 3 markers. As such, multivariate redox profiling could become a useful component of fitness assessment and training optimization in equine athletes.

Our study had some limitations. Its observational nature and lack of a non-exercising control group limit conclusions regarding causality. Additionally, only before and after exercise measurements were collected at each time point; intermediate time points or longitudinal follow-up might have identified more detailed biomarker dynamics. Sample size, although typical for physiological studies in horses, may not capture the full spectrum of inter-individual variation. Although PCA and HC suggested post-exercise redox coordination within this cohort, the study was not powered to exclude small sex- or breed-specific effects. Given published demographic differences in oxidative markers, generalizability is limited and future stratified designs are warranted. Finally, although ELISA kits were validated for equine serum, minor assay variability could have influenced results, although intra- and inter-assay coefficients remained within acceptable limits.

### Conclusions

Within this single-yard cohort of race-trained horses, structured training and racing were associated with a post-exercise reorganization of the redox profile, manifested as stable coupling among SOD/TAOC core with context-dependent positioning of TBARS, while AOPP remained relatively independent. Baseline (pre-exercise) concentrations did not deviate outside of clinically accepted ranges across time points. These findings extend and strengthen existing evidence for training-related redox adaptation using multivariate tools that capture inter-marker coordination beyond univariate changes. We view ratio-type “imbalance indices” and multivariate structure as complementary perspectives on the same biology. Generalizability beyond this cohort should be made with caution. Larger and stratified studies are needed to determine how breed and sex modulate the degree of redox coordination and to test performance-anchored links to conditioning.

## Supplementary Material

aalaf085_Supplemental_Files

## Off-label antimicrobial declaration

The authors declare no off-label use of antimicrobials.

## Institutional animal care and use committee or other approval declaration

This research used the samples collected during standard veterinary procedures. Thus, it does not fall under the legislation for the protection of animals used for scientific purposes, national decree law (Dz. U. 2015 poz. 266 and 2010-63-EU directive). No ethical approval was needed.

## Abbreviations

AOPP: advanced oxidation protein products
CI: confidence interval
ELISA: enzyme-linked immunosorbent assay
FC: fold change
HC: hierarchical clustering
IQR: interquartile range
MANOVA: multivariate analysis of variance
NOx: nitric oxide metabolites
PCA: principal component analysis
RNS: reactive nitrogen species
ROS: reactive oxygen species
R: race (high-intensity exercise condition)
SOD: superoxide dismutase
TAOC: total antioxidant capacity
TBARS: thiobarbituric acid reactive substances
T1: training session 1 (early training phase)
T2: training session 2 (after training adaptation)
TOS: total oxidant status
