# Supplementary material for: Training and race-induced coordination of oxidative stress markers in racehorses: insights from multivariate and univariate analyses
Source: J Vet Intern Med. 2026 Feb 3;40(1):aalaf085. doi: 10.1093/jvimsj/aalaf085 (PMC12866905; doi:10.1093/jvimsj/aalaf085)
Supplement: aalaf085_Supplemental_Files [file aalaf085_supplemental_files.zip › Supplementary_1_stress_NEW_aalaf085.docx]

# Supplementary Figures


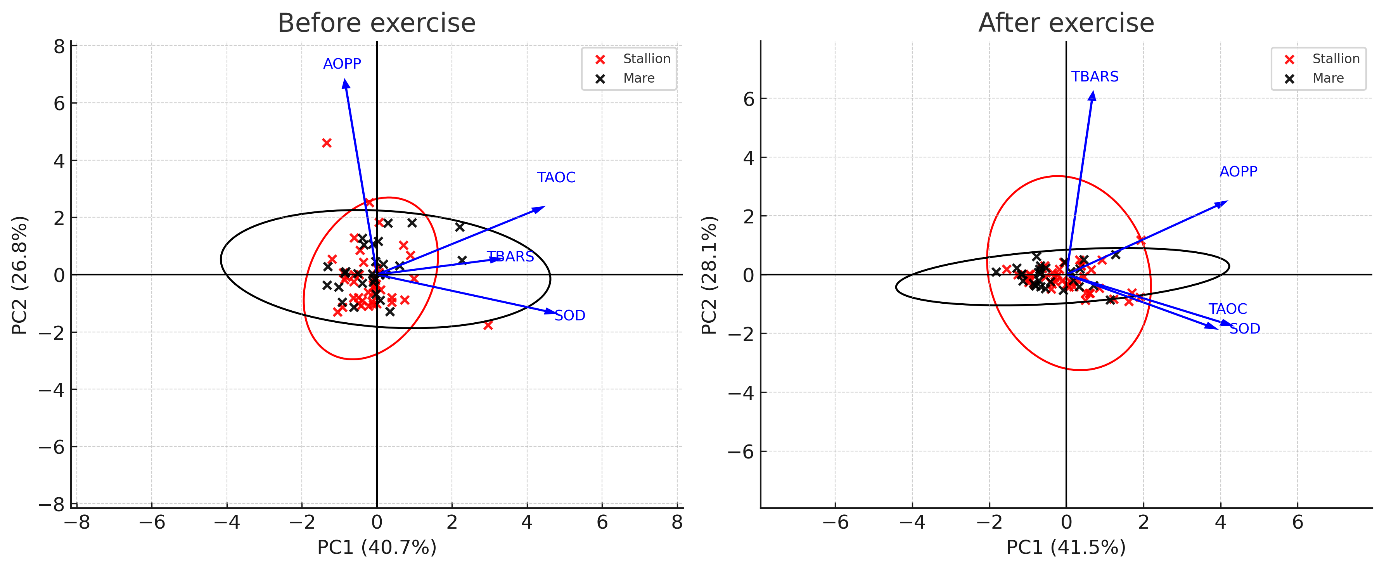


**Supplementary 1 Figure 1. Principal Component Analysis (PCA) of oxidative stress markers presenting sex differences before (a) and after (b) exercise.** PCA biplots illustrate the distribution and relationships of oxidative stress markers—advanced oxidation protein products (AOPP), superoxide dismutase (SOD), total antioxidant capacity (TAOC), and thiobarbituric acid reactive substances (TBARS)—across two principal components (PC1 and PC2). Arrows indicate marker loadings. Red crosses represent stallions, and black crosses represent mares horses. Ellipses depict the 95% confidence intervals for each sex group.

**
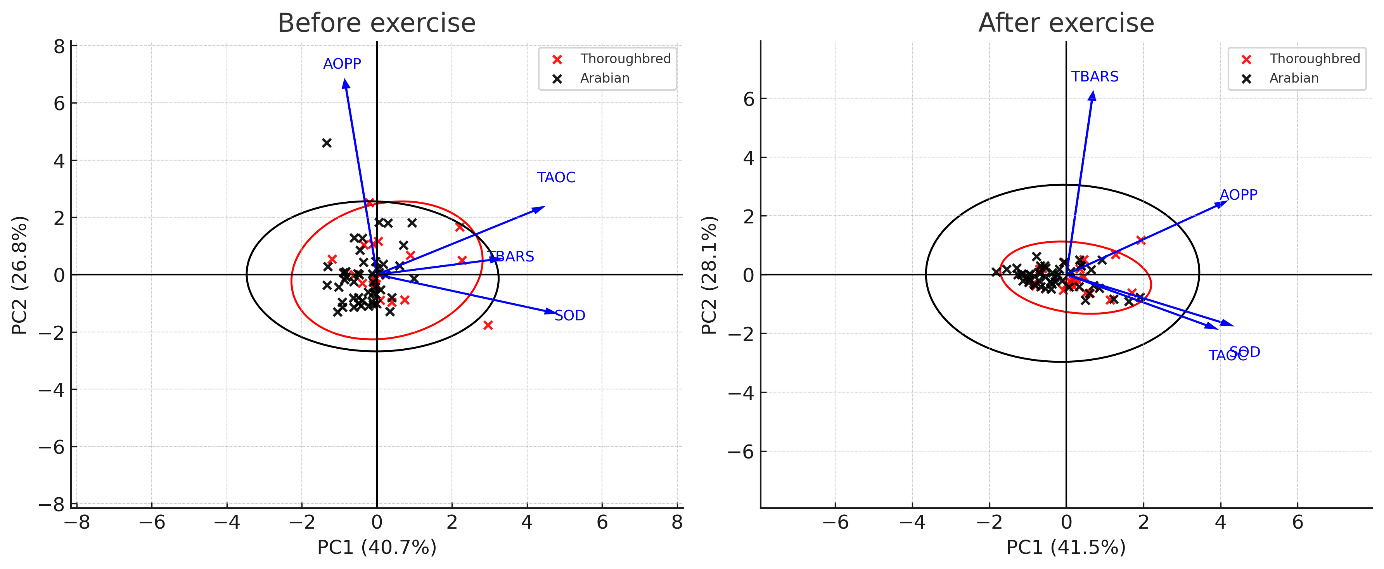
**

**Supplementary 1 Figure 2. Principal Component Analysis (PCA) of oxidative stress markers presenting breed differences before (a) and after (b) exercise.** PCA biplots illustrate the distribution and relationships of oxidative stress markers—advanced oxidation protein products (AOPP), superoxide dismutase (SOD), total antioxidant capacity (TAOC), and thiobarbituric acid reactive substances (TBARS)—across two principal components (PC1 and PC2). Arrows indicate marker loadings. Red crosses represent Thoroughbred horses, and black crosses represent Arabian horses. Ellipses depict the 95% confidence intervals for each breed group.

**
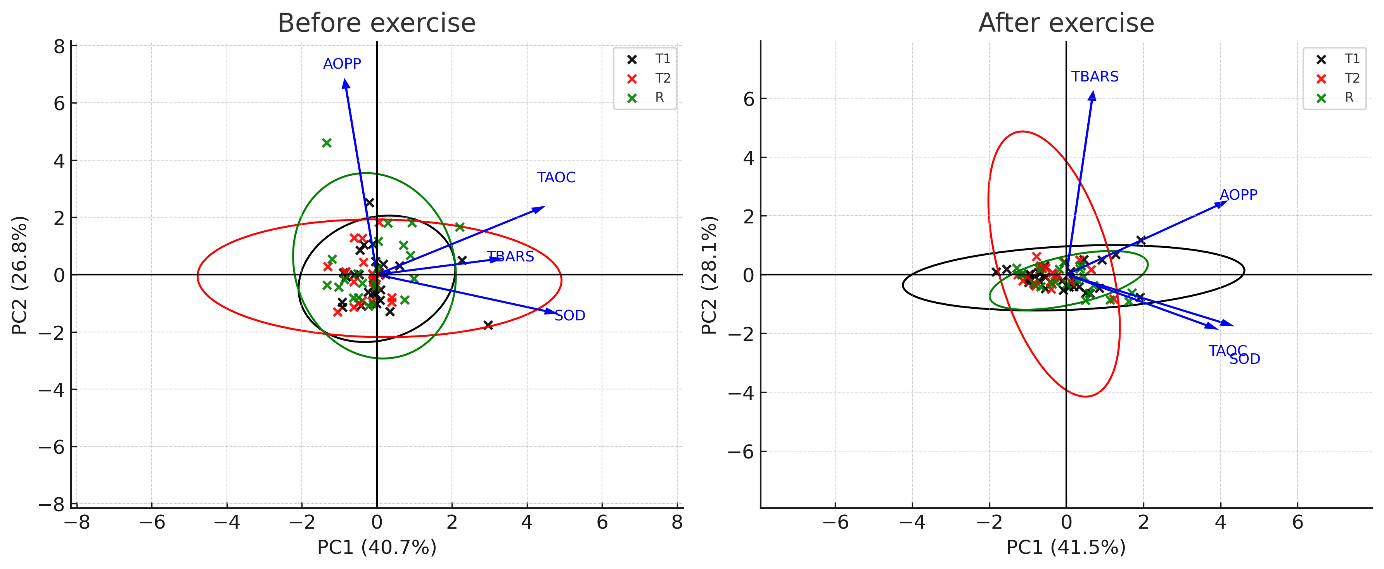
**

**Supplementary 1 Figure 3. Principal Component Analysis (PCA) of oxidative stress markers before (a) and after (b) exercise sessions.** PCA biplots illustrate the distribution of oxidative stress markers—advanced oxidation protein products (AOPP), superoxide dismutase (SOD), total antioxidant capacity (TAOC), and thiobarbituric acid reactive substances (TBARS)—across two principal components (PC1 and PC2). Arrows represent loadings of each marker. Black crosses represent horses of first training session (T1), red crosses second training session (T2), and green crosses the competitive race event (R). Colored ellipses correspond to the 95% confidence intervals for each group.
